# Comparison of Intrathecal Morphine Versus Erector Spinae Plane Block for Perioperative Analgesia in Patients Undergoing Lumbar Spine Surgery: A Randomized Control Trial

**DOI:** 10.7759/cureus.64775

**Published:** 2024-07-17

**Authors:** Arpita Lal, Manish K Singh, Shashank Kumar Kanaujia, Neel Kamal Mishra, Brijesh Pratap Singh, Gyan Prakash Singh

**Affiliations:** 1 Anesthesiology, King George's Medical University, Lucknow, IND; 2 Anesthesiology and Critical Care, King George's Medical University, Lucknow, IND

**Keywords:** numeric rating scale, erector spinae plane block, intrathecal morphine, spine surgeries, low backache

## Abstract

Background: Lumbar spine surgery is associated with a significant degree of moderate-to-severe perioperative pain which can be alleviated by using different pain-relieving modalities; of these, erector spinae plane block and intrathecal morphine have promise.

Purpose: To compare the analgesic efficacy between intrathecal morphine (ITM) versus erector spinae plane block (ESPB) for perioperative analgesia in patients undergoing lumbar spine surgery.

Method: A total of 74 patients aged between 20 and 65 years of either sex were posted for elective lumbar spine surgery. Patients were divided into two groups: Group A patients received 0.3 mg intrathecal morphine and Group B received bilateral erector spinae plane block at L3 level by using 30 mL of 0.5% ropivacaine before starting the surgery for perioperative analgesia. In the perioperative period, pain was assessed by hemodynamic parameters (heart rate and mean arterial pressure), numeric rating scale (NRS), and patient satisfaction score.

Result: The difference in heart rate and mean arterial pressure was found to be statistically significant between groups at three, six, 12, and 24 hours (p<0.05). The patients who required rescue analgesia in Group A and Group B were 23 (62.2%) and 37 (100%) patients in the first 24 hours. The rate of complication was higher in Group A than in Group B (45% vs 5.4%). The patient satisfaction score was found to be better in Group A than in Group B.

Conclusion: Intrathecal morphine provides more substantial and extended analgesia up to 48 hours postoperatively as compared to erector spinae plane block.

## Introduction

Lumbar spine surgery can be categorized into simple surgical interventions like microsurgical discectomy and/or decompression, as well as complex interventions such as fusions and disc prostheses at three or more levels or scoliosis correction surgery. However, lumbar spine surgery is associated with a significant degree of moderate-to-severe perioperative pain. Adequate perioperative pain management can help in early postoperative ambulation, early discharge, and improved long-term outcomes [1]. The role and efficacy of multimodal analgesia (MMA) have already been advocated for in the literature, but insufficient evidence did not provide clear guidelines for combinations of various analgesic modalities [2]. Regional anesthesia is one of the important components of multimodal analgesia [3,4].

The erector spinae plane block (ESPB), an innovative regional anesthetic technique, has garnered significant attention in recent times. ESPB was initially demonstrated to mitigate shingles-related pain, in which local anesthetics (LA) and adjuvants are deposited between the transverse process of vertebrae and erector spinae muscle, and exerts its effects on the ventral and dorsal rami of the spinal nerves by diffusing cranially and caudally [5]. Sono anatomy is easily discernible, and no structures in proximity are susceptible to needle puncture [6,7]. By inhibiting the ESP, bladder and motor neuron function are maintained, enabling prompt mobilization. As motor function remains unaffected, the neurological function of the spinal cord can be assessed promptly following the procedure. Recent case studies [8-11] demonstrate that an ESP block effectively alleviates pain for a variety of indications, including vertebral metastases, lumbar transverse process fractures, lumbar spine fusion, and scoliosis surgery. ESPB's efficacy in spinal procedures is the subject of recent randomized controlled trials (RCTs); however, the results are inconsistent [6].

The preliminary documentation of intrathecal morphine (ITM) administrations was published in 1979 [12]. Morphine’s hydrophilic nature allows it to bind strongly to central nervous system opioid receptors, ensuring prolonged presence in the water-soluble cerebrospinal fluid, thereby extending its analgesic effects up to 24 hours and has emerged as a viable option for perioperative pain management in patients undergoing lumbar spine surgery. Due to ambiguities about the optimal analgesic dosage and duration and documented side effects such as pruritus, respiratory depression, and nausea and vomiting, ITM continues to be encircled by several uncertainties and controversies about its effectiveness in the context of lumbar spine surgeries [13-15]. Throughout history, there have been apprehensions regarding the reliability and simplicity of access to the thecal sac [16-18]. However, ITM proves cost-effective by promoting early mobilization and reducing hospital stays.

We hypothesized that ITM is more effective than ESPB for postoperative analgesia following lumbar spine surgery.

## Materials and methods

**Figure 1:**
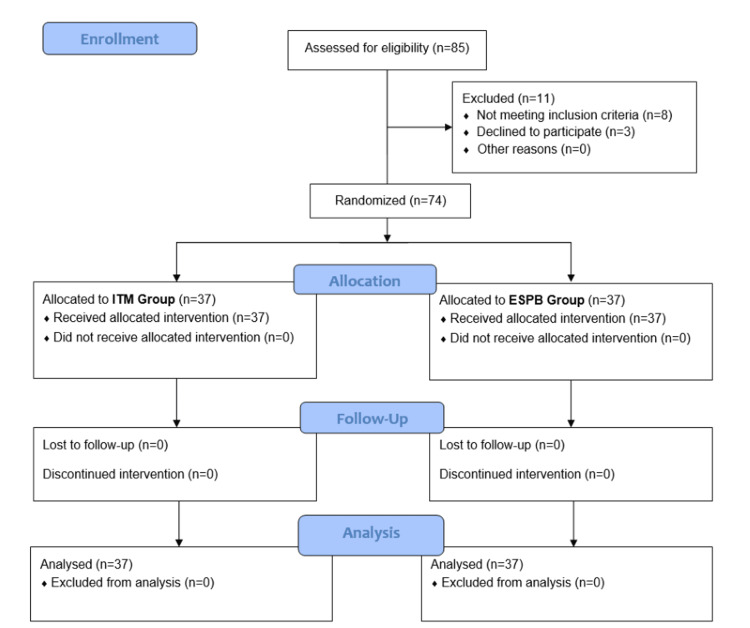
Consort flow diagram

This prospective, single-blind, randomized controlled trial was conducted in the neurosurgery operation theatre of King George's Medical University, Lucknow after approval by the institutional ethics committee (Ref. code: XV-PGTSC-ⅡA/P43). A total of 74 patients of either sex and aged between 20-65 years, having ASA I/II status, scheduled for elective lumbar spine surgery at Levels 1 or 2, were enrolled in the present study after obtaining written informed consent. Patients who refused, as well as those with coagulation disorder, allergies to study drugs, revision lumbar surgeries, severe respiratory illness (eg. chronic obstructive pulmonary disease and obstructive sleep apnea), pregnancy or lactation, chronic opioid, or analgesic use/abuse were excluded. This study was conducted from September 2023 to May 2024. The present study was registered with the Clinical Trial Registry of India with CTRI No. CTRI/2023/09/057847 (Figure 1).

The primary objective of this study was to compare the analgesia using the Numeric Rating Scale (NRS) in ITM versus erector spine plane block for perioperative analgesia in patients undergoing lumbar spine surgery.

All patients were randomly assigned to either Group A or Group B using a computer-generated random table. Group A received ITM, whereas Group B underwent ultrasound-guided ESPB for perioperative analgesia. A preoperative assessment of all the patients was conducted. Patients were instructed on the use of the NRS for pain assessment. General anesthesia was given as per institutional protocol using propofol, midazolam, fentanyl, and vecuronium for induction and anesthesia was maintained by oxygen, air, sevoflurane, and vecuronium infusion. For perioperative pain, ITM and ESPB were used as per group allotted.

In Group A, ITM was administered before induction as 0.3 mg morphine in 2 ml 0.9% normal saline (NS) by the index anesthesiologist under aseptic conditions. Patients were positioned in a sitting posture, and a spinal puncture was performed at the L3-L4 interspace using a 25-gauge Whitacre pencil point spinal needle. Upon confirmation of free cerebrospinal fluid flow, ITM 0.3 mg in 2 ml normal saline 0.9% was administered, following which the patient was repositioned in a supine position.

In Group B (erector spinae plane block), the drug preparation consisted of 30 mL of 0.5% ropivacaine with a total maximum dose of 3 mg/kg. Patients were placed in the prone position, and the spine was palpated upwards from L4-L2, with the L3 position marked on the skin. After ensuring skin asepsis, a high-frequency linear ultrasound probe, enclosed in a sterile sheath, was longitudinally positioned 3 cm lateral to the L3 spinous process. The quadratus lumborum and erector spinae muscles were identified, and the skin was infiltrated with a local anesthetic. A 20-G Quincke needle was then inserted using an in-plane cephalad to caudal direction approach, with the tip placed into the fascial plane on the deep (anterior) aspect of the erector spinae muscle. Confirmation of the needle tip location was done by visible fluid spread below the erector spinae muscle off the bony shadow of the transverse process. A total volume of 15 ml of 0.5% ropivacaine was injected through the needle, and the procedure was repeated on the opposite side. Continuous monitoring of electrocardiography and oxygen saturation was conducted, with baseline heart rate and non-invasive blood pressure recorded before the block, and subsequently every five minutes for 30 minutes. Any block-related complications, such as hypotension or vascular puncture, were diligently documented.

Hemodynamic parameters (heart rate [HR] and mean arterial pressure [MAP]) were observed (at baseline, after intubation, at prone, 30 minutes, 1 hour, end of the surgery, post-op 1, 2, 3, 6, 12, and 24 hours) in the perioperative period. The severity of pain and level of sedation were assessed using NRS and Ramsay sedation score respectively at the time of extubation and then at intervals of one, two, three, six, 12, and 24 hours. Patient satisfaction was observed by using the Patient Satisfaction Scale in the postoperative period at the end of 24 hours; patients were assessed for the quality of pain relief on a four-point pain satisfaction scale, with 1 indicating excellent, 2 indicating very good, 3 indicating satisfactory, and 4 indicating poor. Patients with NRS scores> 4 were managed by giving rescue analgesia in the order of paracetamol (10-15 mg/kg IV), diclofenac sodium (1-2 mg/kg IM/IV) and tramadol (1-2 mg/kg IV) respectively.

Complications such as PONV, pruritis, urinary retention, local site hematoma, oxygen desaturation, and constipation were noted.

Statistical analysis

The data were entered into Microsoft Excel and analyzed utilizing SPSS version 25.0 (IBM Corp., Armonk, NY). Continuous variables were expressed as mean (standard deviation) or range as necessary. Dichotomous variables were represented by number/frequency and analyzed using the chi-square or Fisher's exact test. Student's t-test was employed to compare the means between the two groups. A significance level of p < 0.05 was considered statistically significant.

## Results

The mean age of patients in Group ITM was 40.08 years with a standard deviation (SD) of 12.81, while in Group ESPB, it was 36.03 years with an SD of 13.11. In Group ITM, there were 15 (40.54%) males and 22 (59.46%) females, whereas in Group ESPB, there were 17 (45.95%) males and 20 (54.05%) females. Demographic data of both groups were comparable having no statistically significant difference.

Pain score was found to be higher all the time in Group ESPB in comparison to Group ITM but a statistically significant difference was found at 3, 6, 12, and 24 hours post-surgery (p > 0.001) (Table 1).

**Table 1:** Distribution of studied patients based on the Numeric rating scale in both groups

Numeric Rating Scale	Group ITM (n=37)	Group ESPB (n=37)	p-value
T0 (at extubation)	0.56±0.8	0.81±1.0	0.281
1 hour	1.1±0.8	1.3±1.2	0.401
2 hour	1.2±0.7	1.4±0.8	0.256
3 hour	1.2±0.8	2.5±0.7	<0.001
6 hour	1.3±0.7	3.0±0.9	<0.001
12 hour	1.4±0.6	3.8±1.2	0.001
24 hour	3.2±1.1	4.9±1.3	0.001

HR was found to be statistically significantly higher in Group ESPB in comparison to Group ITM at T8, T9, T10, and T11, with statistical significance (Table 2).

**Table 2:** Distribution of studied patients based on heart rate in both groups

Heart rate	Group ITM (n=37)	Group ESPB (n=37)	p-value
T0 (baseline)	86.2±15.8	81.9±10.1	0.166
T1 (post-intubation)	89.7±18.8	91.0±9.4	0.345
T2 (prone)	89.9±10.1	91.4±9.0	0.521
T3 (at 30 minutes)	84.4±12.2	82.9±9.9	0.576
T4 (1 hour)	85.3±11.1	81.5±8.7	0.105
T5 (end of surgery)	93.2±14.2	89.1±9.3	0.143
T6 (post-op 1 hour)	89.6±13.4	84.5±11.5	0.084
T7 (2 hours)	88.6±9.5	85.2±9.1	0.120
T8 (3 hours)	84.2±11.2	89.9±10.2	0.025
T9 (6 hours)	88.6±9.9	93.9±11.6	0.038
T10 (12 hours)	95.3±10.8	101.3±8.9	0.011
T11 (24 hours)	101.4±12.6	108.6±7.9	0.004

MAP was found to be statistically significantly higher in Group ESPB in comparison to Group ITM at T8, T9, T10, and T11 (p<0.05) (Table 3).

**Table 3:** Distribution of studied patients based on MAP in both groups MAP: mean arterial pressure

MAP	Group ITM (n=37)	Group ESPB (n=37)	p-value
T0 (baseline)	96.1±6.6	98.7±6.2	0.085
T1 (post-intubation)	97.9±6.7	100.2±5.2	0.103
T2 (prone)	97.6±8.9	100.2±5.1	0.129
T3 (at 30 minutes)	94.8±8.0	97.5±5.6	0.096
T4 (1 hour)	93.9±8.6	96.7±4.5	0.083
T5 (end of surgery)	103.7±7.2	105.8±6.2	0.183
T6 ( post-op 1 hour)	96.2±5.7	98.5±6.1	0.098
T7 (2 hours)	97.5±4.5	99.3±5.6	0.131
T8 (3 hours)	98.0±5.6	102.0±5.7	0.003
T9 (6 hours)	99.4±4.8	103.6±4.9	0.004
T10 (12 hours)	101.1±4.1	104.5±5.6	0.003
T11 (24 hours)	99.8±4.2	102.8±5.3	0.008

The patients in the ITM group had a higher Ramsay Sedation Scale score in comparison to the patients in the ESPB group, which was statistically significant (p <0.001) (Table 4).

**Table 4:** Distribution of studied patients based on Ramsay Sedation Scale score in both groups

	Ramsey Sedation Scale Score		
Time	Group ITM (n=37)	Group ESPB (n=37)	p-value
T5 (at extubation)	3.1±0.8	2.2±0.9	<0.001
T6 (1 hour post extubation)	2.8±0.5	2.1±1.1	<0.001
T7 (2 hours)	2.6±0.6	2.1±0.9	<0.001
T8 (3 hours)	2.7±0.9	1.9±0.7	<0.001
T9 (6 hours)	2.4±0.7	1.8±0.6	<0.001
T10 (12 hours)	2.2±0.5	1.5±0.5	<0.001
T11 (24 hours)	2.0±0.4	1.2±0.2	<0.001

Among the patients in the ITM group, 23 (62.2%) patients required rescue analgesia. In contrast, all patients (100%) in the ESPB group required rescue analgesia in the first 24 hours. The difference in the need for rescue analgesia between the two groups was statistically significant (p<0.05) (Table 5).

**Table 5:** Distribution of studied patients based on total rescue analgesia in both groups

Parameters	Group ITM (n=37)	Group ESPB (n=37)	p-value
Need for rescue analgesia	23/37 (62.2%)	37/37 (100.0%)	<0.001
No. of times rescue analgesia was required			
1	10 (27.0%)	0	
2	13 (35.2%)	18 (48.6%)	
3	0	17 (45.9%)	
4	0	2 (5.4%)	

**Figure 2:**
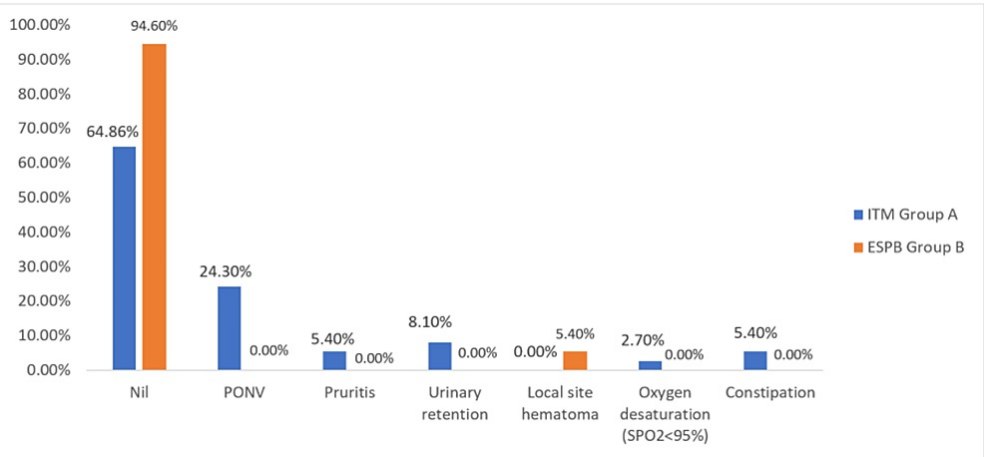
Distribution of complications in both groups

In this study, the ITM group had more incidence of complications than the ESPB group and it was statistically significant (p<0.001) (Figure 2).

**Figure 3:**
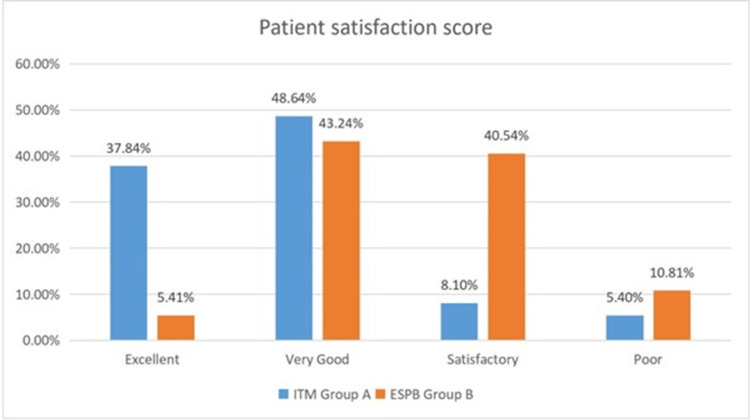
Patient satisfaction score at the end of 24 hours post-surgery

In the present study patients in the ITM group had better satisfaction scores than the ESPB group at the end of 24 hours post-surgery, which was statistically significant (p <0.001) (Figure 3).

## Discussion

In the present study, at the time of extubation (T0) and one hour post-surgery, Group ESPB displayed non-significantly higher pain scores (p > 0.05). This trend continued and it became statistically significant at three, six, 12, and 24 hours post-surgery (p < 0.001). Our findings were supported by Mahmoud et al., who reported that the postoperative VAS scores were significantly lower in the ITM group than the ESPB group throughout all the recorded postoperative study time points until 48 hours postoperative (p < 0.001) [6]. Sawi and Choy reported significantly lower pain scores in their morphine group up to 20 hours postoperatively [11]. Similarly, Kong et al. reported that a small dose of ITM (0.2 mg) produced excellent postoperative analgesia for the first 48 hours after laparoscopic colorectal surgery [7]. The efficacy of 0.4 mg of ITM in delivering postoperative analgesia in patients undergoing posterior interbody fusion operations was shown in a paper by Ziegeler et al. [19].

In this study at the point of prone positioning (T2), and at one hour (T4), the heart rate in Group ESPB was higher, as compared to Group ITM and this was statistically non-significant. It was seen that Group ESPB consistently showed higher heart rates compared to Group ITM at T8, T9, T10, and T11, which was statistically significant. After the prone position (T2) and T4, T5, T6, and T7, Group ESPB consistently displayed a higher MAP compared to Group ITM, which was non-significant. In three hours, post-surgery (T8) marked a particularly significant difference, which was higher in group ESPB. The divergence continued further, up to 24 hours post-surgery (T9, T10, T11); the difference was statistically significant (p = 0.05).

Our findings were consistent with the findings of Mahmoud et al., who reported that a nonsignificant difference was found between the two groups considering intraoperative heart rate until two hours postoperative (p > 0.05), except at 30 minutes (p = 0.022) [6]. Nevertheless, the heart rate was significantly lower in the ITM group compared to the ESPB group in all study postoperative time points except 24 hours (p = 0.045). A significant difference was found regarding the mean arterial blood pressure readings immediately postoperatively, four hours postoperatively, and 24 hours postoperatively (p < 0.05). At the same time, there were non-significant differences at the remaining postoperative times (p > 0.05) [6]. Postoperative hemodynamic indicators (diastolic and systolic blood pressure, and MAP) were sustained within normal ranges in a study conducted by El Sherif et al., with no significant alterations in patients undergoing laparoscopic bariatric surgery (p > 0.05) [9].

In this study difference in the mean Ramsey sedation score was statistically significant at all times up to 24 hours between the two groups (p < 0.001). According to Mahmoud et al., a significant difference was found between the two groups regarding the postoperative Ramsay Sedation Scale score in the first six hours postoperatively (p < 0.05), while a nonsignificant difference was observed at the remaining study time points (p > 0.05) [6]. On the other hand, Kara et al. reported that sedation was similar between their study groups [10]. The difference between our results and theirs may be that they compared ITM with intravenous patient-controlled analgesia with morphine continued for 48 hours, which might increase the sedation score and mask any variation in sedation between the groups. 

In our study, for the first 24 hours, rescue analgesia was required for 23 (62.2%) patients in Group ITM. In contrast, all patients in Group ESPB required rescue analgesia. In Group ITM, 10 (27.0%) patients required rescue analgesia only once, while 13 (35.2%) required rescue analgesia twice. On the other hand, in Group ESPB, 18 patients (48.6%) needed rescue analgesia twice, 17 (45.9%) required it three times, and two (5.4%), four times. The differences in the distribution of rescue analgesia between the two groups were statistically significant (p < 0.001). Sayed et al. reported that nalbuphine and ketorolac consumption over time were significantly different between subjects in both groups [F (1,38) = 25.5 for nalbuphine and 63.5 for ketorolac, p < 0.001]. A significant decrease in nalbuphine and ketorolac consumption was observed in the ITM group when compared to the ESPB group at 0-24 postoperatively [20]. Mahmoud et al. reported that the time to the first rescue analgesia and doses of postoperative analgesia required was significant (p < 0.05) [6].

In this study, 13 (35.14%) patients in Group ITM experienced complications, in contrast to Group ESPB where only two (5.4%) patients encountered complications, which was statistically significant (p < 0.001) between the two groups. Our findings were comparable to the findings of Mahmoud et al. who reported that a higher incidence of complications was recorded in the ITM group (p = 0.001). There were 33 patients (80%) in the ITM group that had at least one complication. The most common complication associated with the use of ITM is urine retention, accounting for approximately 30% of cases [6]. In the same way, a paper by Ruan showed an estimated rate of complications of 42% to 80%, which was increasingly common in elderly patients. Nausea, pruritus, urine retention, vomiting, and constipation were the most common [21].

In Group ITM, 14 patients (37.84%) rated their satisfaction as excellent, 18 (48.64%) as very good, three (8.1%) as satisfactory, and only two patients (5.4%) in Group ITM rated their satisfaction as poor 24 hours post-surgery. In contrast, in Group ESPB, two (5.41%) patients rated their satisfaction as excellent, 16 (43.24%) patients as very good, 15 (40.54%) patients as satisfactory, and four (10.81%) patients rated their satisfaction as poor. The differences in patient satisfaction scores between the two groups were statistically significant (p < 0.001). Our findings were in concordance with the findings of Sawi and Choy who reported that there was a significant difference between the morphine and no morphine groups in terms of overall patient satisfaction and better patients' overall satisfaction in the morphine group [11].

In this study, demographic data were comparable in both groups.

The limitations of our study are its small sample size and single-center structure. A multicenter study with a large sample size might yield additional findings.

## Conclusions

We conclude that intrathecal morphine provides more substantial and extended analgesia (up to 48 hours postoperatively) than an erector spinae plane block, as measured by NRS scores at different time points and doses of postoperative analgesic requirements. There was a significant difference in terms of lower pain score, and higher incidence of side effects with better patients' overall satisfaction in the morphine group.
